# Task‐Evoked Theta Oscillation Dysregulation Underlies Attention Network Deficits in Left Temporal Lobe Drug‐Resistant Epilepsy

**DOI:** 10.1111/cns.70450

**Published:** 2025-06-12

**Authors:** Changqing Zhan, Qiao Wang, Wenyu Wang, Na Pan, Jieju Feng, Zonghan Yang, Shizao Fei, Zongsheng Chen, Yingnian Chen

**Affiliations:** ^1^ Department of Neurology Wuhu No. 2 People's Hospital Wuhu Anhui China; ^2^ Department of Pathology Wuhu No. 2 People's Hospital Wuhu Anhui China; ^3^ Graduate School of Bengbu Medical University Bengbu Anhui China; ^4^ Department of Neurological Rehabilitation Anhui Wannan Rehabilitation Hospital (Wuhu No. 5 People's Hospital) Wuhu Anhui China; ^5^ Graduate School of Wannan Medical College Wuhu Anhui China

**Keywords:** attention network, drug‐resistant epilepsy, neural oscillation, theta

## Abstract

**Aims:**

This study investigated the neural mechanisms of left temporal lobe drug‐resistant epilepsy (DRE) with attention network dysfunction using the attention network test (ANT) and synchronous electroencephalography (EEG).

**Methods:**

This study enrolled three cohorts: 20 patients with left temporal lobe drug‐resistant epilepsy (DRE group), 20 left temporal lobe drug‐sensitive epilepsy (DSE group) patients, and 20 age‐/sex‐matched healthy controls (Ctrl group). Participants completed standardized ANT tasks while scalp EEG was recorded at a 1000 Hz sampling rate. We computed power spectral density (PSD) of neural oscillations from ANT‐task EEG epochs.

**Results:**

DRE patients exhibited significantly impaired network efficiency across executive control (EC), alerting, and orienting networks. Compared to the Ctrl group, both DRE and DSE groups demonstrated reduced frontal theta PSDs in EC, alerting, and orienting networks (all *p* < 0.001), with the DRE group showing greater deficits than the DSE group in the EC network (*p* < 0.001). Additionally, Significant correlations emerged between frontal theta PSD and behavior in Ctrl (EC__effect_: *r* = −0.659, *p* = 0.002; Alerting__effect_: *r* = 0.690, *p* = 0.001; Orienting__effect_: *r* = 0.649, *p* = 0.002) and DSE (EC__effect_: *r* = −0.595, *p* = 0.006; Alerting__effect_: *r* = 0.592, *p* = 0.006; Orienting__effect_: *r* = 0.588, *p* = 0.006). In the DRE group, however, The theta band PSDs of the attention network show no significant correlation with behavioral response effects.

**Conclusion:**

Drug‐resistant epilepsy patients have dysfunctional attention network behavior and decreased theta band PSDs in the frontal lobe. It is possible that decreased theta oscillations in the frontal lobe may contribute to ANT behavior dysfunction in drug‐resistant epilepsy patients.

## Graphical abstract

Using the attention network test (ANT) and task‐state electroencephalography, this study investigated the neural mechanisms of left temporal drug‐resistant epilepsy with attention network dysfunction.

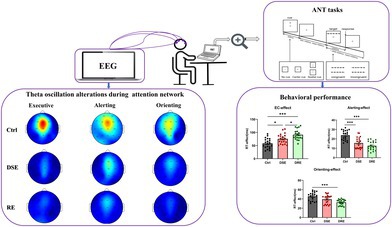

**Abbreviations**
Attention network test: ANT
Executive control: EC

## 1 Introduction

Epilepsy, a chronic neurological disorder manifesting as recurrent unprovoked seizures, affects 70 million individuals globally. With chronic progression and 60%–70% recurrence rates, it constitutes 0.5%–1% of the worldwide disease burden [1]. Low‐and middle‐income countries (LMICs) exhibit significantly higher prevalence and incidence rates of epilepsy compared to high‐income countries (HICs) [2]. China bears an estimated 10 million active epilepsy cases, imposing substantial socioeconomic burdens on both public health systems and household livelihoods [3]. Persistent neuroplastic alterations in individuals with epilepsy drive multifaceted impairments, encompassing neurophysiological abnormalities, cognitive deficits (particularly in executive function, episodic memory, and sustained attention), and psychological comorbidities [4, 5]. Longitudinal cohort studies demonstrate that 34.1% of patients treated with ≥ 2 appropriately selected antiseizure medications (ASMs) fail to achieve seizure freedom for either 12 consecutive months or three times the longest pre‐intervention seizure‐free interval, thus meeting the International League Against Epilepsy (ILAE) criteria for drug‐resistant epilepsy (DRE) [6, 7]. Studies have demonstrated that cognitive impairment in patients with epilepsy is strongly associated with seizure type, age of onset, frequency and severity of seizures, and antiepileptic medications [8, 9]. Uncontrolled seizures disrupt both the functional integrity and structural organization of cortical networks, thereby contributing to cognitive deficits [10, 11]. The intractable nature of DRE is associated with a higher prevalence of cognitive impairment [12, 13].

Cognitive decline initially manifests as heterogeneous impairment across cognitive domains, with deficits in attention, executive function, and short‐term memory often emerging at early stages [14, 15]. Attention is defined as a cognitive process that selectively prioritizes specific stimuli; it encompasses attentional mechanisms involving stimulus prioritization, filtering, and processing [16]. The attention network comprises three functionally and anatomically distinct subnetworks: the executive control (EC), the alerting, and the orienting networks [17]. The Attention Network Test (ANT), developed by Fan et al., concurrently assesses the behavioral efficiency of the three attentional subnetworks: alerting, orienting, and executive control. [18] Neuroimaging studies during attentional tasks consistently identify sustained frontoparietal network (FPN) activation, particularly within the dorsolateral prefrontal cortex (DLPFC) and posterior parietal cortex (PPC), as measured by functional magnetic resonance imaging (fMRI) [19]. Accumulating neurophysiological evidence indicates that attentional networks are characterized by distinct oscillatory patterns [20, 21]. Attentional networks critically rely on theta oscillations (4–8 Hz) for optimal functioning [22, 23]. Patients with left temporal lobe epilepsy (LTLE) exhibit more pronounced alterations in interhemispheric functional connectivity and global structural integrity compared to those with right temporal lobe epilepsy (RTLE) [24]. However, the impact of left temporal lobe drug‐resistant epilepsy (LT‐DRE) on the efficiency of attentional networks and theta‐band (4–8 Hz) oscillatory dynamics has not yet been empirically established.

In this study, we acquired 19‐channel electroencephalography (EEG) signals from patients with LT‐DRE during attentional network behavioral tasks, aiming to investigate whether theta‐band (4–8 Hz) neural oscillations exhibit alterations linked to attentional network dysfunction.

## 2 Subjects and Methods

### 2.1 Study Subjects

We recruited 40 patients with left temporal lobe epilepsy from the outpatient department of the Second People's Hospital of Wuhu. The diagnostic criteria for left temporal lobe epilepsy (LTLE) included: (1) clinical manifestations indicative of an epileptogenic focus localized to the left temporal lobe; (2) neuroimaging evidence (MRI) of left temporal lobe pathology (e.g., focal lesions, hippocampal sclerosis, or atrophy) without extra‐temporal abnormalities; and (3) EEG confirmation of left temporal epileptiform discharges during ictal or interictal phases. DRE was defined by ILAE criteria [6, 7] as meeting all following conditions: (1) Failure to achieve ≥ 12 consecutive months of seizure freedom or three times the longest pre‐intervention seizure‐free interval. (2) Adequate trials of two ASMs with: (a) Appropriate selection matching seizure type and syndrome classification; (b) Optimization of dosage (maximally tolerated or evidence‐based serum concentration); (c) Adherence verification through therapeutic drug monitoring. Twenty patients were classified into each group: DRE and drug‐sensitive epilepsy (DSE). Diagnosis of left‐sided DRE and DSE was established according to ILAE criteria, integrating comprehensive evaluations including ictal semiology, long‐term video‐EEG monitoring, brain MRI findings, and ASM responsiveness, with rigorous exclusion of pseudo‐resistance [6, 7, 25]. The following inclusion criteria were also required: (i) age 16–60 years; (ii) right‐hand dominance with normal or corrected‐to‐normal vision; (iii) mini‐Mental State Examination (MMSE) score > 24; (iv) capacity to provide written informed consent and actively engage in neurocognitive assessments. It was excluded if it met the following one item: (i) comorbid neurological/psychiatric disorders or secondary cognitive impairment; (ii) major systemic comorbidities affecting cardiovascular, cerebral, renal, or hepatic functions; (iii) inability to complete neuropsychological evaluations or comply with experimental protocols.

The DRE group comprised 9 male and 11 female patients with a mean age of 45.30 ± 3.13 years (range: 41–55 years). The median age at onset was 36.5 years (interquartile range: 33–40 years; overall range: 22–45 years). The median disease duration was 9.5 years (interquartile range: 4–12 years; overall range: 1–25 years). Patients had 9–15 years of education with a mean duration of 11.75 ± 1.62 years.

The DSE group comprised 11 male and 9 female patients with a mean age of 45.20 ± 3.37 years (range: 40–54 years). The median age at onset was 34.5 years (interquartile range: 32–40 years; overall range: 20–41 years), and the median disease duration was 11.5 years (interquartile range: 4–14 years; overall range: 3–25 years). Educational attainment ranged from 9 to 15 years, with a mean of 11.60 ± 2.11 years.

Both groups received ASMs, including oxcarbazepine, levetiracetam, sodium valproate, lamotrigine, and topiramate, administered either as monotherapy or combination therapy.

The healthy control group (Ctrl, *n* = 20) comprised volunteers recruited from physical examinations at Wuhu Second People's Hospital. All participants provided written informed consent, and the study protocol was approved by the institutional Ethics Committee.

### 2.2 Behavioral Paradigm

**FIGURE 1:**
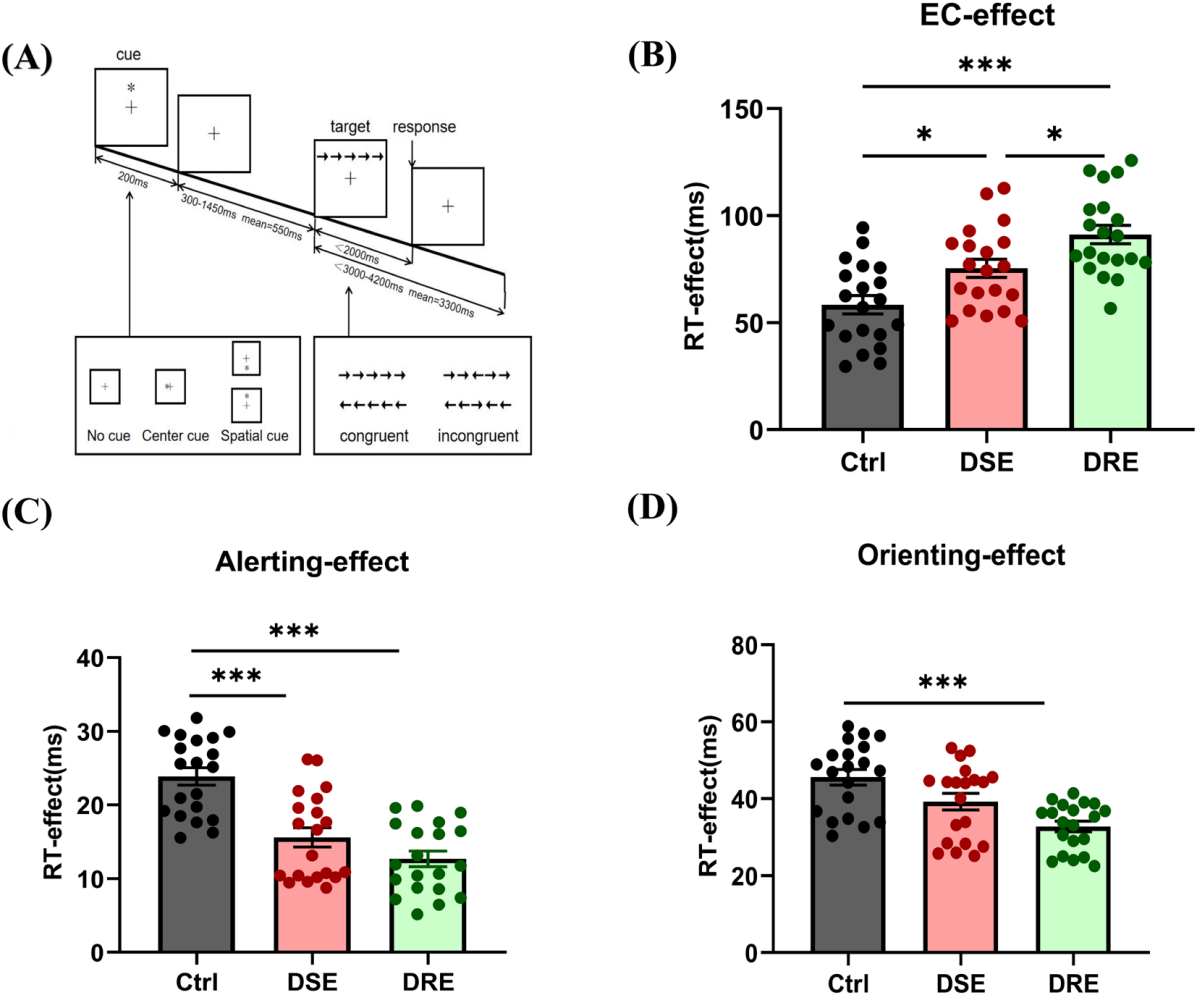
Behavioral performance (A) Attention Network Test. On the screen, the “+” is always in the center. During each trial, the cue is displayed for 200 ms and then disappears. It takes a variable amount of time (300–1450 ms) for the target stimulus (the middle arrow) to appear. Within 2000 milliseconds, participants must respond to the target stimulus before it disappears. There is a variable delay between the appearance of the stimulus and the start of a new trial (3000–4200 ms). Each testing session included 6 practice trials (for protocol acclimation) and 108 formal trials. Participants completed 6 consecutive sessions (2–5 min apart), preceded by clinician‐guided practice (24 trials) to ensure ANT procedural familiarity. (B) EC__effect_ during executive control tasks. (C) Alerting__effect_ during alerting tasks. (D) Orienting__effect_ during orienting tasks. Data presented as mean ± SD. *p < 0.05, **p < 0.01, ***p < 0.001 (one‐way ANOVA with Bonferroni correction).

The ANT, originally developed by Fan [18] and adapted using E‐Prime 2.0 (Figure 1A) as described in prior studies [22, 23, 26] was employed in this study. During the ANT task, a fixation cross (“+”) was displayed centrally. Each trial consisted of five horizontally aligned black arrows. The central arrow (target) pointed either left or right and was flanked by two arrows on each side, which were either congruent (same direction as the target) or incongruent (opposite direction). Participants were instructed to indicate the target direction by pressing the “1” key (left) or “2” key (right) within 2000 ms. A pre‐target asterisk (“*”) was presented to facilitate rapid stimulus detection. Three cue conditions were included: no cue, central cue, and spatial cue. Each participant completed six blocks with 108 experimental trials and six buffer trials per block, interspersed with 2–5 min breaks. Trials with incorrect responses or reaction times (RT) outside the 200–1000 ms range were excluded from analysis.

EC [27] reflects the ability to resolve cognitive conflicts. The EC effect (EC__effect_) is defined as the difference in reaction time (RT) between incongruent trials (RT__incongruent_) and congruent trials (RT__congruent_), calculated as:

(1) $${\mathrm{EC}}_{\_\text{effect}}={\mathrm{RT}}_{\_\text{incongruent}}-{\mathrm{RT}}_{\_\text{congruent}}$$

A smaller EC__effect_ indicates superior executive control function. Reaction times for incongruent and congruent trials are denoted as RT__incongruent_ and RT__congruent_, respectively.

The alerting network [27] represents the capacity to sustain optimal vigilance during task performance. The alerting effect (Alerting__effect_) is defined as the difference in RT between trials with no cue (RT__no_) and trials with a center cue (RT__center_), calculated as:

(2) $${\text{Alerting}}_{\_\text{effect}}={\mathrm{RT}}_{\_\mathrm{no}}-{\mathrm{RT}}_{\_\text{center}}$$

RT__no_ and RT__center_ denote reaction times during no‐cue and center‐cue trials, respectively. A higher Alerting___effect indicates enhanced alerting function.

Orienting [27], a cognitive process of attending to spatial information, involves the selection and processing of environmental cues. The orienting effect (calculated as response time in center‐cue trials minus response time in spatial‐cue trials) quantifies the efficiency of spatial attention allocation. This metric was derived using the following formula:

(3) $${\text{Orienting}}_{\_\text{effect}}={\mathrm{RT}}_{\_\text{center}}-{\mathrm{RT}}_{\_\text{spatial}}$$

Where RT__center_ and RT__spatial_ represent the mean response times (ms) in the center cue condition and spatially predictive cue condition, respectively. Increased orienting__effect_ values indicate enhanced orienting function.

All participants underwent the ANT during morning sessions. For patients in the DRE and DSE groups, testing was conducted prior to morning ASMs administration, with confirmation of seizure‐free status during the assessment period.

### 2.3 Recording of Electroencephalograms

Neural activity during the ANT was acquired using a Nicolet EEG system (Model YZB/USA 2783‐2011; Natus Medical Incorporated, Pleasanton, CA). Continuous EEG signals were recorded from 19 Ag/AgCl electrodes (Fp1, Fp2, F7, F3, Fz, F4, F8, T3, C3, Cz, C4, T4, T5, P3, Pz, P4, T6, O1, O2) positioned according to the international 10–20 system with extended coverage. Data were sampled at 1000 Hz with all electrode impedances maintained below 5 kΩ throughout recordings.

### 2.4 Electroencephalogram Analysis

#### 2.4.1 Neural Oscillations' Time‐Frequency Distribution

Time‐frequency local window functions are selected for the short‐time Fourier transform (STFT). Within a short time interval, the analysis window function *g*(*t*) is assumed to be stable (pseudo‐stationary). Stationary signals with finite time widths can be represented by *f*(*t*)*g*(*t*). Each time point's power spectrum is calculated, and the power spectrums are connected to compute the time‐frequency distribution diagram. In this case, we define the STFT of the signal *x*(*t*) as:

(4) $$\text{STFT}\left(f,t\right)=\int_{-\infty }^{+\infty }\left[x\left(t\right)g\left(t-\tau \right)\right]e^{-j2\mathrm{\pi f\tau}}\mathrm{d\tau}$$

Where *g(t)* represents the window function. In this study, the window width was 256 ms and the moving step length was 64 ms.

#### 2.4.2 Neuronal Oscillations' Spatial Distribution

The effective time period of the executive control network is from 300 ms before the response to 300 ms after the response [22, 23]. It is estimated that the effective time period of the alerting and orienting network is 50 ms before a cue appears and 450 ms after a cue appears [23]. Neural oscillations were analyzed across the 0.05–100 Hz spectrum, encompassing five canonical frequency bands: delta (0.05–4 Hz), theta (4–8 Hz), alpha (8–12 Hz), beta (15–30 Hz), and gamma (30–100 Hz). We calculated the 19‐channel EEG's energy spectrum using STFT and extracted the PSD for each channel. By averaging the 19‐channel PSD of correctly responding trials, we were able to draw the PSD of each frequency band on the 19‐channel spatial distribution map. This frequency band was defined as the characteristic frequency band based on the PSD of the frequency band with the highest PSD. A total of 19 channels were identified in four regions: frontal region (Fp1, Fp2, F3, Fz, and F4); parietal region (C3, Cz, C4, P3, Pz, and P4); occipital region (O1, O2) and temporal region (F7, F8, T3, T4, T5, and T6). In each brain area, the PSD of the characteristic frequency band has been calculated. It was determined that the region of the brain with the greatest PSD was the dominant region. The following formula was used to calculate the power of the neural oscillations in the EC networks:

(5) $${\mathrm{PSD}}_{\_\mathrm{EC}}={\mathrm{PSD}}_{\_\text{incongruent}}-{\mathrm{PSD}}_{\_\text{congruent}}$$

(6) $${\mathrm{PSD}}_{\_\text{Alerting}}={\mathrm{PSD}}_{\_\text{center}}-{\mathrm{PSD}}_{\_\mathrm{no}}$$

(7) $${\mathrm{PSD}}_{\_\text{orienting}}={\mathrm{PSD}}_{\_\text{spatial}}-{\mathrm{PSD}}_{\_\text{center}}$$

Where PSD__EC_ denotes the power of the EC network, PSD__incongruent_ denotes the power of incongruent tasks, PSD__congruent_ denotes the power of congruent tasks, PSD__alerting_ denotes the power of the alerting network, PSD__center_ denotes the power of center cue tasks, PSD__no_ denotes the power of no cue tasks, PSD__orienting_ denotes the power of the orienting network, and PSD__spatial_ denotes the power of spatial cue tasks.

### 2.5 Statistical Analysis

All statistical analyses were performed using SPSS 20.0 (SPSS Inc., Chicago, IL). Categorical variables (clinical count data) are presented as frequencies and percentages, with between‐group comparisons performed using Chi‐squared tests or Fisher's exact test as appropriate. For continuous variables, the normality of data distribution was systematically assessed using the Kolmogorov–Smirnov test. Normally distributed data are expressed as mean ± standard deviation (SD) and were analyzed using independent samples *t*‐tests for two‐group comparisons. Non‐normally distributed data are presented as median (IQR, Interquartile Range) with between‐group comparisons performed using the Mann–Whitney *U* test (two groups) following confirmation of non‐normal distribution. For multi‐group comparisons of behavioral performance, one‐way analysis of variance (ANOVA) was employed after verifying normality assumptions, with Bonferroni‐corrected post hoc tests for pairwise comparisons. This analytical approach was similarly applied to comparisons of five frequency bands and four brain regions across groups. Normally distributed neuropsychological parameters were assessed for correlations with frontal theta‐band PSD values during EC, alerting, and orienting tasks using Pearson's correlation analysis. All statistical tests were two‐tailed with *α* = 0.05. Probability values are denoted as *p < 0.05, **p < 0.01, and ***p < 0.001.

## 3 Result

### 3.1 Demographic and Clinical Characteristics Analysis

The demographic and clinical characteristics of healthy controls (Ctrl, *n* = 20), drug‐resistant epilepsy (DRE, *n* = 20), and drug‐sensitive epilepsy (DSE, *n* = 20) are summarized in Table 1.

**TABLE 1:** Group demographics.

Items	Ctrl (20)	DRE (20)	DSE (20)	*χ* ^2^/*F*/Z	df	*p*
Demographics						
Age (year)	40–52 (45.60 ± 3.17)	41–55 (45.30 ± 3.13)	40–54 (45.20 ± 3.37)	*F* = 0.083	2	0.920
Male, *n* (%)	12 (60)	9 (45)	11 (55)	*χ* ^2^ = 0.938	2	0.626
Female, *n* (%)	8 (40)	11 (55)	9 (45)	—	—	—
Education (year)	9–17 (11.65 ± 2.30)	9–15 (11.75 ± 1.62)	9–15 (11.60 ± 2.11)	*F* = 0.028	2	0.972
Clinical features						
MMSE scores	26–30 (27.80 ± 0.95)	25–30 (27.10 ± 1.65)	25–30 (27.50 ± 1.32)	*F* = 1.378	2	0.260
Age of onset (years)	—	36.5 (33, 40)	34.5 (32, 40)	*Z* = −0.462	—	0.644
Duration (years)	—	9.5 (4, 12)	11.5 (4, 14)	*Z* = −0.91	—	0.363
Etiology, *n* (%)						
Traumatic brain injury	—	5 (25)	6 (30)	χ^2^ = 0.422	3	0.936
Stroke	—	4 (20)	5 (25)			
Encephalitis	—	4 (20)	3 (15)			
Unknown	—	7 (35)	6 (30)			
Treatment						
Number of ASMs	—	2 (2, 3)	2 (1, 3)	*Z* = −0.976	—	0.329
ASM types, *n* (%)						
Oxcarbazepine	—	2 (10%)	1 (5%)	χ^2^ = 0.360	1	0.548
Levetiracetam	—	14 (70%)	15 (75%)	χ^2^ = 0.125	1	0.723
Sodium valproate	—	18 (90%)	15 (75%)	χ^2^ = 1.558	1	0.212
Lamotrigine	—	12 (60%)	10 (50%)	χ^2^ = 0.404	1	0.525
Topiramate	—	2 (10%)	1 (5%)	χ^2^ = 0.360	1	0.548
Epilepsy surgery, *n* (%)	—	0	0	—	—	—
Seizure characteristics						
Seizure type, *n* (%)						
Focal	—	13 (65%)	16 (80%)	χ^2^ = 1.129	1	0.288
Generalized	—	7 (35%)	4 (20%)			
Monthly seizure frequency	—	9 (6, 16)	1 (0, 1)	*Z* = −5.432	—	< 0.001

*Note:* ASM, Anti‐seizure medication, among these are oxcarbazepine, levetiracetam, sodium valproate, lamotrigine, and Topiramate; Ctrl, Healthy controls; df, degrees of freedom; DRE, Drug‐resistant epilepsy; DSE, Drug‐sensitive epilepsy; MMSE, Mini‐Mental State Examination.

#### 3.1.1 Demographics

Age ranges and mean values were comparable across groups: Ctrl (40–52 years, mean ± SD: 45.60 ± 3.17), DRE (41–55 years, 45.30 ± 3.13), and DSE (40–54 years, 45.20 ± 3.37) (*F* = 0.083, df = 2, *p* = 0.920). No significant differences were observed in gender distribution (male: 60% [*n* = 12] in Ctrl vs. 45% [*n* = 9] in DRE vs. 55% [*n* = 11] in DSE; *χ* ^2^ = 0.938, df = 2, *p* = 0.626) or education years (Ctrl: 9–17 years, 11.65 ± 2.30; DRE: 9–15 years, 11.75 ± 1.62; DSE: 9–15 years, 11.60 ± 2.11; *F* = 0.028, *p* = 0.972).

#### 3.1.2 Clinical Features

Mini‐Mental State Examination (MMSE) scores showed no intergroup differences (Ctrl: 26–30, 27.80 ± 0.95; DRE: 25–30, 27.10 ± 1.65; DSE: 25–30, 27.50 ± 1.32; *F* = 1.378, *p* = 0.260). Age of onset (median [IQR]: 36.5 [28, 29, 30, 31, 32, 33, 34, 35] in DRE vs. 34.5 [28, 29, 30, 31, 32, 33, 34, 35, 36] in DSE; *Z* = −0.462, *p* = 0.644) and disease duration (9.5 [4, 5, 6, 7, 8, 9, 10, 11, 12] years in DRE vs. 11.5 [4, 5, 6, 7, 8, 9, 10, 11, 12, 13, 14] years in DSE; *Z* = −0.91, *p* = 0.363) were similar between epilepsy groups.

#### 3.1.3 Etiology

Etiological distributions did not differ significantly between DRE and DSE groups (χ^2^ = 0.422, df = 3, *p* = 0.936). Traumatic brain injury (25% [*n* = 5] in DRE vs. 30% [*n* = 6] in DSE), stroke (20% [*n* = 4] vs. 25% [*n* = 5]), encephalitis (20% [*n* = 4] vs. 15% [*n* = 3]), and unknown causes (35% [*n* = 7] vs. 30% [*n* = 6]) were the primary etiologies.

#### 3.1.4 Treatment

The median number of ASMs was comparable between DRE (2 [IQR: 2–3]) and DSE (2 [IQR: 1–3]) groups (*Z* = −0.976, *p* = 0.329). Sodium valproate was the most prescribed ASM (90% [*n* = 18] vs. 75% [*n* = 15]; χ² = 1.558, *p* = 0.212), followed by Levetiracetam (70% [*n* = 14] in DRE vs. 75% [*n* = 15] in DSE; χ^2^ = 0.125, *p* = 0.723). Other ASMs, including oxcarbazepine (10% [*n* = 2] vs. 5% [*n* = 1]), lamotrigine (60% [*n* = 12] vs. 50% [*n* = 10]), and topiramate (10% [*n* = 2] vs. 5% [*n* = 1]), showed no significant differences (all *p* > 0.05). No patients in either group underwent epilepsy surgery.

#### 3.1.5 Seizure Characteristics

Focal seizures predominated in both DRE (65% [*n* = 13]) and DSE (80% [*n* = 16]) groups (χ^2^ = 1.129, *p* = 0.288). Generalized seizures occurred in 35% (*n* = 7) of DRE and 20% (*n* = 4) of DSE cases. Monthly seizure frequency was significantly higher in DRE (median [IQR]: 9 [6, 7, 8, 9, 10, 11, 12, 13, 14, 15, 16]) compared to DSE (1 [0–1]; *Z* = −5.432, *p* < 0.001).

### 3.2 Behavioral Performance

#### 3.2.1 Executive Control Network

Significant group differences in EC__effect_ were observed (one‐way ANOVA: *F* (2.57) = 14.73, *p* < 0.001). Post hoc Bonferroni tests revealed: Elevated EC__effect_ in DRE (91.18 ± 19.26 ms) and DSE (75.47 ± 19.00 ms) groups compared to Ctrl (58.39 ± 19.05 ms; DRE vs. Ctrl: *p* < 0.001; DSE vs. Ctrl: *p* = 0.02). The DRE group showed higher EC__effect_ than the DSE group (Δ = 15.71 ms, *p* = 0.04). (Figure 1B).

#### 3.2.2 Alerting Network

The alerting__effect_ demonstrated significant group variation (*F* (2.57) = 23.72, *p* < 0.001): Attenuated alerting__effect_ in DRE (12.69 ± 4.75 ms) and DSE (15.59 ± 5.88 ms) relative to Ctrl (23.89 ± 5.32 ms; both *p* < 0.001); Non‐significant difference between DRE and DSE (Δ = −2.9 ms, *p* = 0.274) (Figure 1C).

#### 3.2.3 Orienting Network

Group effects reached significance (*F* (2.57) = 11.47, *p* < 0.001): DRE exhibited reduced orienting__effect_ vs. Ctrl (32.79 ± 6.15 ms vs. 45.60 ± 9.14 ms, *p* < 0.001). DSE showed intermediate values (39.27 ± 9.6 ms) with no significant differences from Ctrl (*p* = 0.064) or DRE (*p* = 0.056). (Figure 1D).

### 3.3 The Attention Network's Neural Oscillation Patterns

**FIGURE 2:**
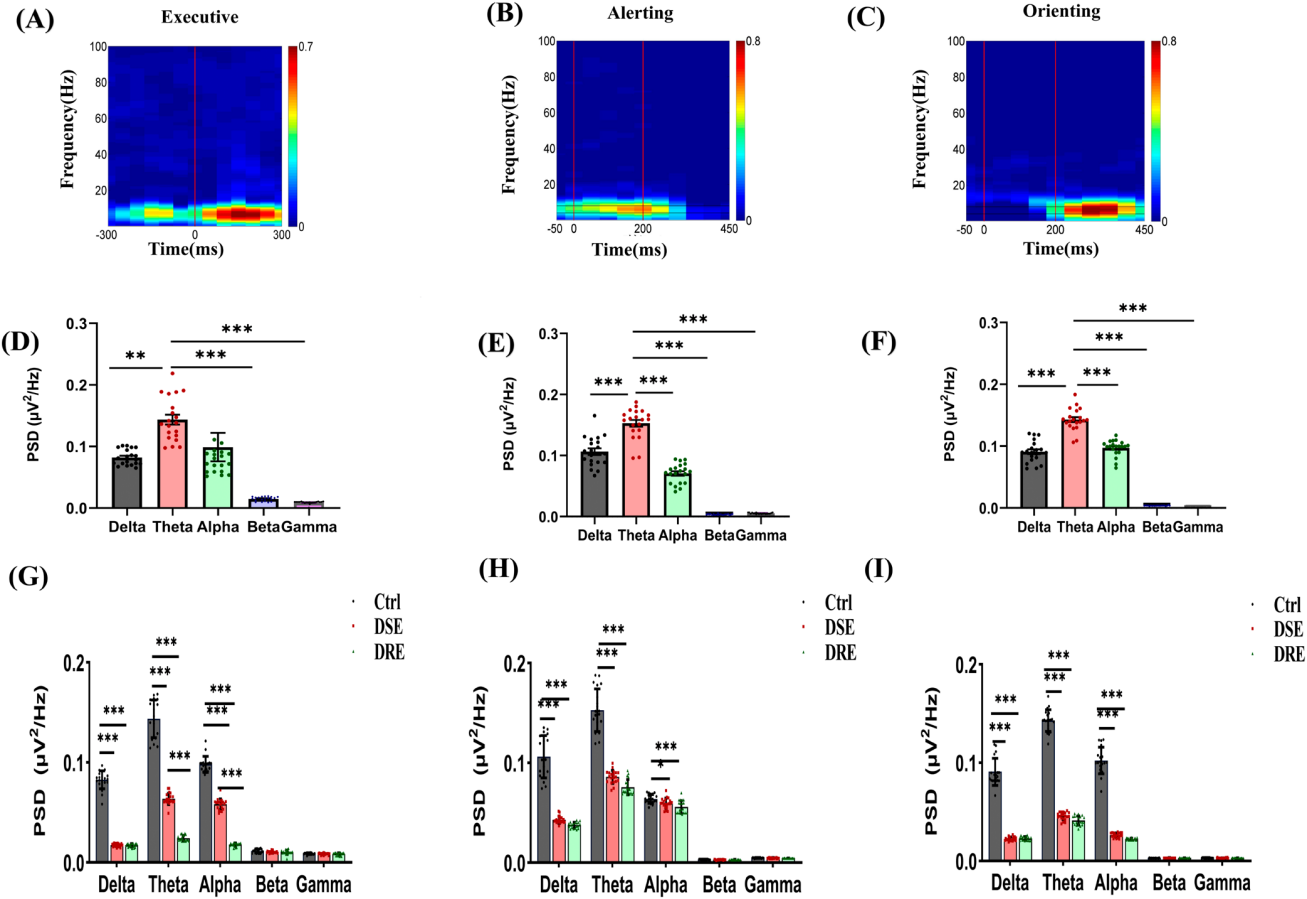
PSD distributions across frequency bands during ANT. (A–C) Time‐frequency representations of PSD in the Fz channel during EC, alerting, and orienting tasks. Axes: Time (x‐axis), frequency (y‐axis). The response onset is marked by a vertical red line, with color intensity representing power magnitude. For EC network analysis, a 256‐ms sliding window (64‐ms step) was applied from 300 ms pre‐response to 300 ms post‐response. For alerting and orienting networks, the window spanned 50 ms pre‐cue to 450 ms post‐cue [22, 23]. (D–F) Whole‐brain averaged PSDs of five frequency bands (delta, theta, alpha, beta, gamma) during each task. Theta band exhibited significantly higher power compared to delta, beta, and gamma bands (Bonferroni‐corrected, all *p* < 0.01). (G–I) Intergroup comparisons of whole‐brain averaged PSDs (Ctrl, DSE, DRE groups) across tasks. Significance levels: *p < 0.05, **p < 0.01, ***p < 0.001.

Power spectrum analysis was performed to identify characteristic frequency bands of attention networks. As shown in Figure 2A–C, the time‐frequency distributions of PSDs in the Fz channel of the control group revealed predominant theta band activity (4–8 Hz) across EC, alerting, and orienting networks. Figure 2D–F demonstrates significant differences in PSDs across five frequency bands (delta, theta, alpha, beta, and gamma) within these networks (one‐way ANOVA, *p* < 0.001). Post hoc Bonferroni tests indicated that theta band PSDs in EC, alerting, and orienting networks were significantly higher than those in delta, beta, and gamma bands (*p* < 0.01). Moreover, theta band PSDs in alerting and orienting networks exceeded those in the alpha band (*p* < 0.001). These findings establish theta band activity as the characteristic frequency signature for EC, alerting, and orienting networks.

Figure 2G–I compares the average PSDs of five frequency bands across Ctrl, DSE, and DRE groups, revealing significant intergroup differences (one‐way ANOVA, *p* < 0.001). Post hoc Bonferroni tests demonstrated that during EC tasks, the whole‐brain PSDs of delta, theta, and alpha bands in both DRE and DSE groups were significantly lower than those in the Ctrl group (*p* < 0.001). Notably, the theta and alpha band PSDs in the DRE group were further reduced compared to the DSE group during EC tasks (*p* < 0.001; Figure 2G). Similar patterns were observed during alerting tasks: DRE and DSE groups exhibited significantly decreased delta, theta, and alpha band PSDs relative to the Ctrl group (*p* < 0.05), though no significant differences emerged between DRE and DSE groups (all *p* > 0.05; Figure 2H). During orienting tasks, both DRE and DSE groups again showed lower delta, theta, and alpha band PSDs than the Ctrl group (all *p* < 0.001), with no statistical differences between the two experimental groups (all *p* > 0.05; Figure 2I).

### 3.4 Characteristic Spatial Distribution Patterns of Neural Oscillations in Attention Network

**FIGURE 3:**
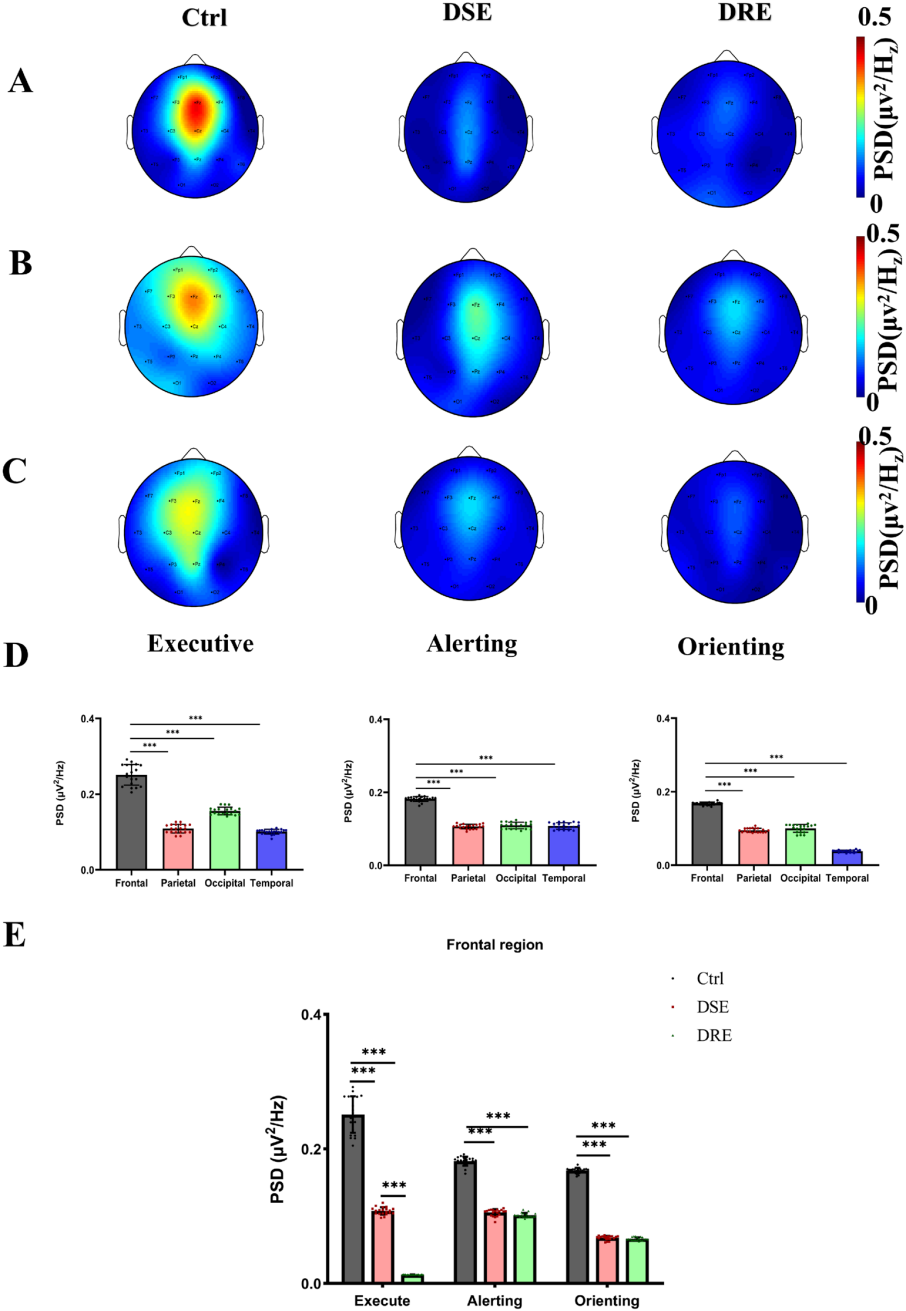
Theta‐band PSD spatial distributions and regional comparisons across attention networks. (A) Theta‐band PSD topographic map in the EC network. (B) Theta‐band PSD topographic map in the alerting network. (C) Theta‐band PSD topographic map in the orienting network. (D) Theta‐band PSD comparisons among four brain regions (frontal, parietal, occipital, temporal) within EC, alerting, and orienting networks. (E) Intergroup comparisons of frontal theta‐band PSDs (Ctrl, DSE, DRE groups) across EC, alerting, and orienting networks. Significance levels: ***p < 0.001.

Figure 3A–C presents the power spectral density topographic maps of the EC, alerting, and orienting networks. The theta‐band neural oscillations predominantly localized to the frontal midline regions, as revealed by the PSD topographic distributions (Figure 3A–C). (D) Regional comparisons of theta‐band PSD across four brain areas (frontal, parietal, occipital, temporal) within each network. Significant interregional differences were observed (one‐way ANOVA, *p* < 0.001). Post hoc Bonferroni tests revealed that frontal theta PSDs exceeded those in parietal, occipital, and temporal regions across all networks (all *p* < 0.001). (E) Intergroup comparisons of frontal theta PSDs (Ctrl vs. DSE vs. DRE groups) within each network. Significant group differences were detected (ANOVA, all *p* < 0.001). During EC tasks, both DRE and DSE groups exhibited reduced frontal theta PSDs compared to Ctrl (Bonferroni, all *p* < 0.001), with DRE showing further reductions relative to DSE (*p* < 0.001). Similar suppression patterns were observed in alerting and orienting networks for DRE/DSE versus Ctrl (all *p* < 0.001), though no significant differences emerged between DRE and DSE groups in these networks (alerting network: *p* = 0.087; orienting network: *p* = 0.344).

### 3.5 Correlation Analysis Between Theta Oscillations and ANT Performance

**FIGURE 4:**
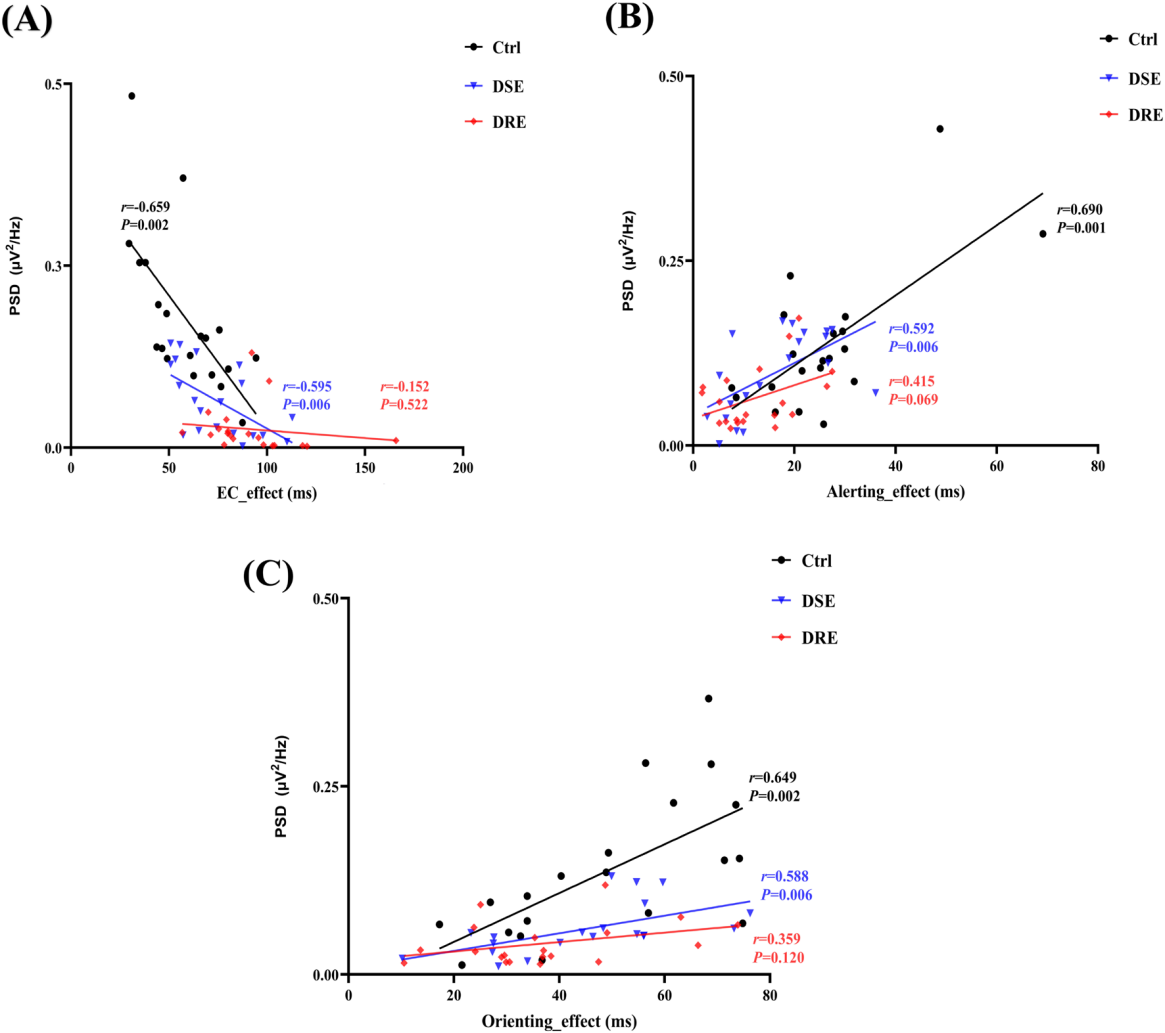
Correlation between theta band PSDs and performance on the ANT tasks. (A) The correlation between theta band PSDs of the frontal region and the EC__effect_; (B) the correlation between theta band PSDs of the frontal region and the Alerting__effect_; (C) the correlation between theta band PSDs of the frontal region and the Orienting__effect_.

Pearson correlation analysis revealed significant associations between ANT performance and theta‐band PSD values in the frontal region across experimental groups (Figure 4A–C). In the EC network, frontal theta PSD demonstrated strong negative correlations with EC__effect_ in both Ctrl (*r* = −0.659, *p* = 0.002) and DSE (*r* = −0.595, *p* = 0.006) groups (Figure 4A). No significant correlation was observed in the DRE group (*r* = −0.152, *p* = 0.522). Positive correlations emerged between frontal theta PSD and Alerting__effect_ for Ctrl (*r* = 0.690, *p* = 0.001) and DSE (*r* = 0.592, *p* = 0.006) groups (Figure 4B). The DRE group showed no statistically significant association (*r* = 0.415, *p* = 0.069). In the orienting network, significant positive correlations were maintained in Ctrl (*r* = 0.649, *p* = 0.002) and DSE (*r* = 0.588, *p* = 0.006) groups regarding Orienting__effect_ (Figure 4C). The DRE group again exhibited a non‐significant correlation (*r* = 0.359, *p* = 0.120).

## 4 Discussion

Our study collected 19‐channel EEG signals from patients with drug‐resistant epilepsy during an attention network behavioral test. We analyzed the patients' attention network function, characterized neural oscillation patterns within the attention network, and investigated dynamic changes in these patterns. Existing evidence demonstrates that enhanced executive control functions are associated with lower EC__effect_ values, whereas improved alerting and orienting functions correlate with higher Alerting__effect_ and Orienting__effect_ magnitudes, respectively [23, 37]. Patients with DSE and DRE exhibited significantly higher EC__effect_ values than the Ctrl group. Notably, the DRE group demonstrated a greater EC__effect_ than the DSE group. These findings suggest progressively impaired executive control functions, with DRE patients showing more pronounced deficits than DSE patients. Both Alerting__effect_ and Orienting__effect_ were significantly reduced in the DRE group compared to the Ctrl group, while the DSE group also displayed diminished Alerting__effect_ relative to the Ctrl group. These findings suggest that both DRE and DSE patients exhibit impaired alerting functions. Moreover, DRE patients demonstrate more pronounced orienting dysfunction compared to the Ctrl group. Theta oscillations dominated all three attention subnetworks (EC, alerting, and orienting) across groups [38, 39]. Compared to controls, both DSE and DRE groups exhibited significantly reduced theta PSDs in the whole‐brain EC, alerting, and orienting networks. Notably, DRE patients showed greater theta PSD attenuation in the EC network than DSE patients, indicating a progressive deterioration of theta‐rhythmic coordination with disease severity. Midline frontal theta oscillations are recognized as a neural hallmark of attention networks and working memory [23, 36, 40]. Our findings extend these observations by demonstrating the frontal cortex serves as the core hub for all three subsystems—EC, alerting, and orienting networks—with spatial localization patterns (Figure 3A–C) corroborating prior neuroimaging evidence [22, 23, 40]. Frontal theta PSDs in EC, alerting, and orienting networks were significantly reduced in both DRE and DSE patients compared to controls. Notably, DRE patients demonstrated greater frontal theta PSD attenuation in the EC network than DSE patients, indicating progressive deterioration of frontal theta rhythmicity with disease severity. These findings establish a hierarchical pattern of frontocortical network dysfunction—where both epilepsy subtypes show impaired theta synchronization across all attention networks, while DRE exhibits selectively enhanced EC network disruption. Based on Pearson correlation analysis, our results showed that in the EC network, theta band PSDs in the frontal region of both Control and DSE groups were negatively correlated with the EC__effect_. In the alerting and orienting networks, theta band PSDs in the frontal region of these groups were positively correlated with Alerting__effect_ and Orienting__effect_, respectively. No significant associations were found in the DRE group, possibly due to significantly weakened theta band PSDs across all three ANT subnetworks in this group. These findings suggest that attenuated theta oscillations in ANT subnetworks may contribute to altered behavioral performance in patients with drug‐resistant epilepsy. Specifically, decreased theta oscillations in the frontal region may represent an underlying neurophysiological mechanism for ANT network deficits observed in left temporal lobe drug‐resistant epilepsy.

High cognitive performance depends on attention, and attentional difficulties have been reported in patients with temporal lobe epilepsy [28, 37]. Hippocampal sclerosis causes cognitive impairment in these patients, which gradually worsens as seizures accumulate and the disease progresses [29, 30]. Drug‐resistant epilepsy, characterized by poor response to antiepileptic drugs and frequent seizures, is associated with more severe cognitive impairment [31]. In this study, patients with left temporal lobe drug‐resistant epilepsy demonstrated prolonged EC__effect_, while Orienting__effect_ and Alerting__effect_ were shortened. These findings indicate impaired behavioral performance across all three ANT subnetworks in left temporal lobe drug‐resistant epilepsy, consistent with previous studies [32, 33]. Neural oscillations represent common electrical activity of neurons in the brain and can be recorded by EEG. These oscillations play a critical role in maintaining information exchange between different brain regions [34, 35]. Theta oscillations (4–8 Hz) are an intrinsic feature of attention networks, driving top‐down neural activity [38, 41, 42]. Recent studies have demonstrated that midfrontal theta oscillations play a dominant role in attentional processes [22, 38, 43]. Our findings indicate that theta band activity is the characteristic neural oscillation pattern in all three attention subnetworks, with frontal regions primarily responsible for these subnetworks. Epileptic seizures disrupt theta oscillations, with the emergence of interictal spikes being a direct cause of theta rhythm disruption [44, 45]. Consistent with previous research, our epilepsy patients exhibited reduced theta oscillations in the frontal region across all three attention subnetworks. Moreover, theta oscillations in the EC network were significantly weaker in patients with drug‐resistant epilepsy compared to those with drug‐sensitive epilepsy.

Previous studies have shown that theta band neural oscillation intensity significantly correlates with cognitive and behavioral performance [46, 47]. In our study, theta band PSDs in both the normal control group and drug‐sensitive epilepsy patients were significantly correlated with behavioral effects across all three attention subnetworks. These findings suggest that theta neural oscillation deficits represent a potential neural mechanism underlying attentional network dysfunction in drug‐resistant patients. Similarly, Pan et al. reported that impairment of working memory in epilepsy patients was associated with alterations in theta neural oscillations [47]. Li et al. found that temporal lobe epilepsy patients exhibit severe executive control functioning deficits, potentially related to frontal theta oscillation abnormalities [40]. Furthermore, our results demonstrated that drug‐resistant epilepsy patients had significantly impaired theta neural oscillations in the executive control network compared to drug‐sensitive epilepsy patients, while orienting and alerting networks showed no significant differences between the two groups. This differential impact may be explained by the distinct neurotransmitter systems regulating these networks [48]. The executive control network is primarily modulated by dopamine, the orienting network by cholinergic systems, and the alerting network by norepinephrine (NE) systems. In drug‐resistant epilepsy, prolonged and frequent seizures can disrupt dopaminergic pathways, reducing striatal dopamine uptake and consequently leading to severe executive control system dysfunction [49, 50, 51].

In temporal lobe epilepsy, structural abnormalities have been documented in multiple brain regions, including the hippocampus, parahippocampal gyrus, amygdala, cingulate gyrus, thalamus, and parietal and prefrontal cortices [52, 53]. These underlying structural alterations may contribute significantly to the pathophysiological mechanisms of temporal lobe epilepsy. Additionally, several clinical factors in temporal lobe epilepsy patients—including age of onset, disease duration, seizure frequency, and antiepileptic medication use—have been associated with cognitive dysfunction. Cognitive impairments are more prevalent in patients with early‐onset epilepsy, longer disease duration, and higher seizure frequency [54]. In drug‐resistant epilepsy, where seizure control remains challenging, recurrent high‐frequency seizures and persistent epileptic discharges disrupt the functional connectivity within and between cortical regions involved in cognitive processing, thereby exacerbating cognitive dysfunction [55].

## 5 Conclusion

In this study, we employed the ANT behavioral test and neural electrophysiological measurements to analyze task‐state oscillations of the attention network in patients with left temporal lobe drug‐resistant epilepsy. Our results demonstrated that these patients exhibited impaired attention network function and decreased theta neural oscillations in the frontal region. Furthermore, we found significant correlations between theta band neural oscillation intensity and the behavioral performance across all three attention subnetworks. These findings suggest that impaired theta neural oscillation patterns may represent a neurophysiological mechanism underlying attention network dysfunction in patients with left temporal lobe drug‐resistant epilepsy.

## Author Contributions

Contributions to the design and concept of this study have been made by all authors. Changqing Zhan and Yingnian Chen designed the study; Wenyu Wang, Qiao Wang, Jieju Feng, Zonghan Yang, Shizao Fei, and Zongsheng Chen collected data; Changqing Zhan and Na Pan analyzed the data; Changqing Zhan drafted the paper. Each author read and approved the final manuscript.

## Ethics Statement

This study was approved by the Ethics Committee of the Second People's Hospital of Wuhu City (approval number: 2024‐KY‐019). Written informed consent was obtained from all participants prior to enrollment. The authors have read the Journal's position on issues involved in ethical publication and affirm that this report is consistent with those guidelines.

## Conflicts of Interest

The authors declare no conflicts of interest.

## Data Availability

The data that support the findings of this study are available on request from the corresponding author. The data are not publicly available due to privacy or ethical restrictions.

## References

[1] E. Akyuz , Y. N. Paudel , A. K. Polat , H. E. Dundar , and E. Angelopoulou , “Enlightening the Neuroprotective Effect of Quercetin in Epilepsy: From Mechanism to Therapeutic Opportunities,” Epilepsy & Behavior 115 (2021): 107701. 33412369 10.1016/j.yebeh.2020.107701

[2] GBD 2016 Neurology Collaborators , “Global, Regional, and National Burden of Neurological Disorders, 1990–2016: A Systematic Analysis for the Global Burden of Disease Study 2016,” Lancet Neurology 18, no. 5 (2019): 459–480. 30879893 10.1016/S1474-4422(18)30499-X PMC6459001

[3] D. Ding , D. Zhou , J. W. Sander , W. Wang , S. Li , and Z. Hong , “Epilepsy in China: Major Progress in the Past Two Decades,” Lancet Neurology 20, no. 4 (2021): 316–326. 33743240 10.1016/S1474-4422(21)00023-5

[4] M. C. de Souza , C. O. Paulo , L. Miyashiro , and C. A. Twardowschy , “Comparison of Screening Tests in the Evaluation of Cognitive Status of Patients With Epilepsy,” Dementia & Neuropsychologia 15, no. 1 (2021): 145–152. 33907608 10.1590/1980-57642021dn15-010016 PMC8049568

[5] C. M. Cornaggia , M. Beghi , M. Provenzi , and E. Beghi , “Correlation Between Cognition and Behavior in Epilepsy,” Epilepsia 47, no. Suppl 2 (2006): 34–39. 17105457 10.1111/j.1528-1167.2006.00685.x

[6] P. Kwan , A. Arzimanoglou , A. T. Berg , et al., “Definition of Drug Resistant Epilepsy: Consensus Proposal by the Ad Hoc Task Force of the ILAE Commission on Therapeutic Strategies,” Epilepsia 51, no. 6 (2010): 1069–1077. 19889013 10.1111/j.1528-1167.2009.02397.x

[7] I. E. Scheffer , S. Berkovic , G. Capovilla , et al., “ILAE Classification of the Epilepsies: Position Paper of the ILAE Commission for Classification and Terminology,” Epilepsia 58, no. 4 (2017): 512–521. 28276062 10.1111/epi.13709 PMC5386840

[8] E. M. Sherman , B. L. Brooks , T. B. Fay‐McClymont , and W. S. MacAllister , “Detecting Epilepsy‐Related Cognitive Problems in Clinically Referred Children With Epilepsy: Is the WISC‐IV a Useful Tool,” Epilepsia 53, no. 6 (2012): 1060–1066. 22554239 10.1111/j.1528-1167.2012.03493.x

[9] S. Auvin , “Paediatric Epilepsy and Cognition,” Developmental Medicine and Child Neurology 64, no. 12 (2022): 1444–1452. 35801543 10.1111/dmcn.15337

[10] Y. Ben‐Ari and G. L. Holmes , “Effects of Seizures on Developmental Processes in the Immature Brain,” Lancet Neurology 5, no. 12 (2006): 1055–1063. 17110286 10.1016/S1474-4422(06)70626-3

[11] H. Yang , C. Zhang , C. Liu , et al., “Brain Network Alteration in Patients With Temporal Lobe Epilepsy With Cognitive Impairment,” Epilepsy & Behavior 81 (2018): 41–48. 29475172 10.1016/j.yebeh.2018.01.024

[12] C. Allone , V. Lo Buono , F. Corallo , et al., “Neuroimaging and Cognitive Functions in Temporal Lobe Epilepsy: A Review of the Literature,” Journal of the Neurological Sciences 381 (2017): 7–15. 28991719 10.1016/j.jns.2017.08.007

[13] E. Heminghyt , H. Herrman , A. H. Skogan , et al., “Cognitive Change After DBS in Refractory Epilepsy: A Randomized‐Controlled Trial,” Acta Neurologica Scandinavica 145, no. 1 (2022): 111–118. 34658033 10.1111/ane.13539

[14] I. M. McDonough , M. M. Wood , and W. S. Miller, Jr. , “A Review on the Trajectory of Attentional Mechanisms in Aging and the Alzheimer's Disease Continuum Through the Attention Network Test,” Yale Journal of Biology and Medicine 92, no. 1 (2019): 37–51. 30923472 PMC6430165

[15] Y. Han , F. Zhang , Y. Tian , P. Hu , B. Li , and K. Wang , “Selective Impairment of Attentional Networks of Alerting in Wilson's Disease,” PLoS One 9, no. 6 (2014): e100454. 24949936 10.1371/journal.pone.0100454 PMC4065050

[16] M. E. le Pelley , C. J. Mitchell , T. Beesley , D. N. George , and A. J. Wills , “Attention and Associative Learning in Humans: An Integrative Review,” Psychological Bulletin 142, no. 10 (2016): 1111–1140. 27504933 10.1037/bul0000064

[17] A. Raz and J. Buhle , “Typologies of Attentional Networks,” Nature Reviews. Neuroscience 7, no. 5 (2006): 367–379. 16760917 10.1038/nrn1903

[18] J. Fan , B. D. McCandliss , T. Sommer , A. Raz , and M. I. Posner , “Testing the Efficiency and Independence of Attentional Networks,” Journal of Cognitive Neuroscience 14, no. 3 (2002): 340–347. 11970796 10.1162/089892902317361886

[19] B. Xuan , M. A. Mackie , A. Spagna , et al., “The Activation of Interactive Attentional Networks,” NeuroImage 129 (2016): 308–319. 26794640 10.1016/j.neuroimage.2016.01.017 PMC4803523

[20] J. Fan , J. Byrne , M. S. Worden , et al., “The Relation of Brain Oscillations to Attentional Networks,” Journal of Neuroscience 27, no. 23 (2007): 6197–6206. 17553991 10.1523/JNEUROSCI.1833-07.2007 PMC6672149

[21] C. He , R. K. Chikara , C. L. Yeh , and L. W. Ko , “Neural Dynamics of Target Detection via Wireless EEG in Embodied Cognition,” Sensors (Basel) 21, no. 15 (2021): 5213. 34372448 10.3390/s21155213 PMC8348206

[22] Y. Ren , L. Pan , X. Du , et al., “Theta Oscillation and Functional Connectivity Alterations Related to Executive Control in Temporal Lobe Epilepsy With Comorbid Depression,” Clinical Neurophysiology 131, no. 7 (2020): 1599–1609. 32417702 10.1016/j.clinph.2020.03.038

[23] D. Guo , C. Zhan , J. Liu , et al., “Alternations in Neural Oscillation Related to Attention Network Reveal Influence of Indoor Toluene on Cognition at Low Concentration,” Indoor Air 32, no. 7 (2022): e13067. 35904384 10.1111/ina.13067

[24] P. Besson , V. Dinkelacker , R. Valabregue , et al., “Structural Connectivity Differences in Left and Right Temporal Lobe Epilepsy,” NeuroImage 100 (2014): 135–144. 24814212 10.1016/j.neuroimage.2014.04.071

[25] J. Zelano , T. Stödberg , and T. Tomson , “Classification of Seizures and Epilepsies,” Läkartidningen 115 (2018): EZ3Z. 29786805

[26] C. Zhan , J. Liu , M. Cui , et al., “Alterations in Functional Connectivity of Executive Control Network Reveal Influence of Indoor Toluene on Cognition at Low Concentration,” Building and Environment 232 (2023): 110031, 10.1016/j.buildenv.2023.110031.

[27] S. E. Petersen and M. I. Posner , “The Attention System of the Human Brain: 20 Years After,” Annual Review of Neuroscience 35 (2012): 73–89. 10.1146/annurev-neuro-062111-150525 PMC3413263 22524787

[28] D. J. Englot , V. L. Morgan , and C. Chang , “Impaired Vigilance Networks in Temporal Lobe Epilepsy: Mechanisms and Clinical Implications,” Epilepsia 61, no. 2 (2020): 189–202. 31901182 10.1111/epi.16423 PMC7033006

[29] L. C. Black , B. K. Schefft , S. R. Howe , J. P. Szaflarski , H. S. Yeh , and M. D. Privitera , “The Effect of Seizures on Working Memory and Executive Functioning Performance,” Epilepsy & Behavior 17, no. 3 (2010): 412–419. 20153981 10.1016/j.yebeh.2010.01.006

[30] S. Gulati , S. Yoganathan , and B. Chakrabarty , “Epilepsy, Cognition and Behavior,” Indian Journal of Pediatrics 81, no. 10 (2014): 1056–1062. 25073691 10.1007/s12098-014-1530-4

[31] A. Gavrilovic , G. Toncev , T. Boskovic Matic , K. Vesic , J. Ilic Zivojinovic , and J. Gavrilovic , “Impact of Epilepsy Duration, Seizure Control and EEG Abnormalities on Cognitive Impairment in Drug‐Resistant Epilepsy Patients,” Acta Neurologica Belgica 119, no. 3 (2019): 403–410. 30737651 10.1007/s13760-019-01090-x

[32] B. Esteso Orduña , M. C. Del Fournier Castillo , S. Cámara Barrio , et al., “Cognitive and Behavioral Profiles of Pediatric Surgical Candidates With Frontal and Temporal Lobe Epilepsy,” Epilepsy & Behavior 117 (2021): 107808, 10.1016/j.yebeh.2021.107808. 33640566

[33] N. Law , E. Widjaja , and M. L. Smith , “Unique and Shared Areas of Cognitive Function in Children With Intractable Frontal or Temporal Lobe Epilepsy,” Epilepsy & Behavior 80 (2018): 157–162. 29414546 10.1016/j.yebeh.2017.12.035

[34] S. R. Cole and B. Voytek , “Brain Oscillations and the Importance of Waveform Shape,” Trends in Cognitive Sciences 21, no. 2 (2017): 137–149. 28063662 10.1016/j.tics.2016.12.008

[35] G. Buzsáki and A. Draguhn , “Neuronal Oscillations in Cortical Networks,” Science 304, no. 5679 (2004): 1926–1929. 15218136 10.1126/science.1099745

[36] L. Pan , J. Wang , W. Wu , Y. Wang , Y. Zhu , and Y. Song , “Transcutaneous Auricular Vagus Nerve Stimulation Improves Working Memory in Temporal Lobe Epilepsy: A Randomized Double‐Blind Study,” CNS Neuroscience & Therapeutics 30, no. 2 (2024): e14395. 37553557 10.1111/cns.14395 PMC10848055

[37] Y. Ren , L. Pan , X. Du , Y. Hou , X. Li , and Y. Song , “Functional Brain Network Mechanism of Executive Control Dysfunction in Temporal Lobe Epilepsy,” BMC Neurology 20, no. 1 (2020): 137. 32295523 10.1186/s12883-020-01711-6 PMC7161158

[38] R. F. Helfrich , A. Breska , and R. T. Knight , “Neural Entrainment and Network Resonance in Support of Top‐Down Guided Attention,” Current Opinion in Psychology 29 (2019): 82–89. 30690228 10.1016/j.copsyc.2018.12.016 PMC6606401

[39] C. Wang , X. Wang , M. Zhu , et al., “Spectrum Power and Brain Functional Connectivity of Different EEG Frequency Bands in Attention Network Tests,” Annual International Conference of the IEEE Engineering in Medicine and Biology Society. IEEE Engineering in Medicine and Biology Society. Annual International Conference 2021 (2021): 224–227. 34891277 10.1109/EMBC46164.2021.9630869

[40] X. Li , Y. Hou , Y. Ren , X. Tian , and Y. Song , “Alterations of Theta Oscillation in Executive Control in Temporal Lobe Epilepsy Patients,” Epilepsy Research 140 (2018): 148–154. 29358157 10.1016/j.eplepsyres.2017.12.017

[41] O. Jensen and L. L. Colgin , “Cross‐Frequency Coupling Between Neuronal Oscillations,” Trends in Cognitive Sciences 11, no. 7 (2007): 267–269. 17548233 10.1016/j.tics.2007.05.003

[42] A. M. Cebolla and G. Cheron , “Understanding Neural Oscillations in the Human Brain: From Movement to Consciousness and Vice Versa,” Frontiers in Psychology 10 (2019): 1930. 31507490 10.3389/fpsyg.2019.01930 PMC6718699

[43] L. T. Hsieh and C. Ranganath , “Frontal Midline Theta Oscillations During Working Memory Maintenance and Episodic Encoding and Retrieval,” NeuroImage 85 (2014): 721–729. 23933041 10.1016/j.neuroimage.2013.08.003 PMC3859771

[44] M. Ge , D. Wang , G. Dong , et al., “Transient Impact of Spike on Theta Rhythm in Temporal Lobe Epilepsy,” Experimental Neurology 250 (2013): 136–142. 24100023 10.1016/j.expneurol.2013.09.023 PMC3857724

[45] X. Fu , Y. Wang , A. N. Belkacem , et al., “Interictal Spike and Loss of Hippocampal Theta Rhythm Recorded by Deep Brain Electrodes During Epileptogenesis,” Sensors (Basel) 22, no. 3 (2022): 1114. 35161860 10.3390/s22031114 PMC8838088

[46] E. Tan , S. V. Troller‐Renfree , S. Morales , et al., “Theta Activity and Cognitive Functioning: Integrating Evidence From Resting‐State and Task‐Related Developmental Electroencephalography (EEG) Research,” Developmental Cognitive Neuroscience 67 (2024): 101404. 38852382 10.1016/j.dcn.2024.101404 PMC11214181

[47] L. Pan , D. Guo , J. Wang , et al., “Alterations in Neural Oscillations Related to Working Memory Deficit in Temporal Lobe Epilepsy,” Epilepsy & Behavior 121 (2021): 108063. 34052633 10.1016/j.yebeh.2021.108063

[48] J. Fossella , M. I. Posner , J. Fan , J. M. Swanson , and D. W. Pfaff , “Attentional Phenotypes for the Analysis of Higher Mental Function,” ScientificWorldJournal 2 (2002): 217–223. 12806053 10.1100/tsw.2002.93 PMC6009361

[49] V. Bouilleret , F. Semah , A. Biraben , et al., “Involvement of the Basal Ganglia in Refractory Epilepsy: An 18F‐Fluoro‐L‐DOPA PET Study Using 2 Methods of Analysis,” Journal of Nuclear Medicine 46, no. 3 (2005): 540–547. 15750171

[50] A. Depaulis and S. L. Moshé , “The Basal Ganglia and the Epilepsies: Translating Experimental Concepts to New Therapies,” Epileptic Disorders 4, no. Suppl 3 (2002): S7–S8. 12495870

[51] C. Deransart , B. T. Lê‐Pham , E. Hirsch , C. Marescaux , and A. Depaulis , “Inhibition of the Substantia Nigra Suppresses Absences and Clonic Seizures in Audiogenic Rats, but Not Tonic Seizures: Evidence for Seizure Specificity of the Nigral Control,” Neuroscience 105, no. 1 (2001): 203–211. 11483312 10.1016/s0306-4522(01)00165-8

[52] L. Chauvière , “Update on Temporal Lobe‐Dependent Information Processing, in Health and Disease,” European Journal of Neuroscience 51, no. 11 (2020): 2159–2204. 31605644 10.1111/ejn.14594

[53] X. Y. Tai , B. Bernhardt , M. Thom , et al., “Review: Neurodegenerative Processes in Temporal Lobe Epilepsy With Hippocampal Sclerosis: Clinical, Pathological and Neuroimaging Evidence,” Neuropathology and Applied Neurobiology 44, no. 1 (2018): 70–90. 29288503 10.1111/nan.12458

[54] O. N. Arski , J. M. Young , M. L. Smith , and G. M. Ibrahim , “The Oscillatory Basis of Working Memory Function and Dysfunction in Epilepsy,” Frontiers in Human Neuroscience 14 (2020): 612024, 10.3389/fnhum.2020.612024. 33584224 PMC7874181

[55] C. E. Elger , C. Helmstaedter , and M. Kurthen , “Chronic Epilepsy and Cognition,” Lancet Neurology 3, no. 11 (2004): 663–672. 15488459 10.1016/S1474-4422(04)00906-8

